# Supplementary material for: Optimization of the Enzymatic Protein Hydrolysis of By-Products from Seabream (Sparus aurata) and Seabass (Dicentrarchus labrax), Chemical and Functional Characterization
Source: Foods. 2020 Oct 20;9(10):1503. doi: 10.3390/foods9101503 (PMC7589672; doi:10.3390/foods9101503)
Supplement: Supplementary file 1 [file foods-09-01503-s001.pdf]

Table S1. Experimental domain and coding of the independent variables in the factorial design executed to study the joint effect of pH and temperature on the Alcalase hydrolysis of seabream and seabass heads.

	**Natural values**	
**Coded values**	**pH**	**T (°C)**
-1.41	6.0	30.0
-1	6.6	37.3
0	8.0	55.0
+1	9.4	72.7
+1.41	10.0	80.0

Codification: $V_c = (V_n - V_0)/\Delta V_n$
Decodification: $V_n = V_0 + (\Delta V_n \times V_c)$
V_n = natural value of the variable to codify
ΔV_n = increment of V_n for unit of V_c
V_0 = natural value in the centre of the domain
V_c = codified value of the variable

Constant conditions
Agitation= 200 rpm
r (S:L)= 1:1
[Alcalase]= 0.5% (v/w) or 12 AU/kg of heads
time of hydrolysis= 3 h

Table S2. Fatty acids content (as % of total fatty acids) in the fish oils recovered from Sb_He, Sb_FT, Sb_Vis, Sbass_He, Sbass_FT and Sbass_Vis, complementary to the production of FPHs. Errors are the confidence intervals for n=2 (samples from independent hydrolysates) and α= 0.05.

Formula	Fatty acids	Sb_He	Sb_FT	Sb_Vis	Sbass_He	Sbass_FT	Sbass_Vis
**C8:0**	Caprylic acid	0.07±0.04	0.08±0.03	0.08±0.02	0.07±0.05	0.17±0.06	0.08±0.02
**C10:0**	Capric acid	0.08±0.02	0.10±0.02	0.11±0.03	0.09±0.02	0.21±0.05	0.09±0.04
**C11:0**	Undecanoic acid	0.04±0.01	0.05±0.02	0.05±0.02	0.04±0.02	0.10±0.03	0.05±0.01
**C12:0**	Lauric acid	0.12±0.04	0.14±0.06	0.16±0.07	0.13±0.08	0.28±0.10	0.15±0.05
**C13:0**	Tridecanoic acid	0.06±0.02	0.07±0.02	0.08±0.03	0.07±0.01	0.13±0.06	0.07±0.02
**C14:0**	Myristic acid	2.42±0.42	2.51±0.28	2.62±0.19	2.76±0.63	2.58±0.57	2.60±0.70
**C14:1**	Myristoleic acid	0.11±0.02	0.10±0.01	0.14±0.05	0.09±0.06	0.20±0.08	0.12±0.05
**C15:0**	Pentadecanoic acid	0.32±0.06	0.34±0.05	0.33±0.07	0.34±0.08	0.31±0.02	0.26±0.07
**C16:0**	Palmitic acid	15.36±0.90	15.21±0.58	15.27±0.49	14.73±1.35	13.22±0.98	13.59±1.01
**C16:1n7c**	Palmitoleic acid	4.45±0.40	4.24±0.35	5.66±0.62	4.59±0.88	4.94±0.47	4.89±0.56
**C17:0**	Heptadecanoic acid	0.52±0.09	0.55±0.08	0.52±0.03	0.55±0.04	0.38±0.05	0.40±0.06
**C18:0**	Stearic acid	2.41±0.23	2.98±0.19	3.06±0.36	3.13±0.62	2.68±0.11	2.59±0.24
**C18:1n9c**	Oleic acid	28.59±0.69	28.68±0.93	37.73±1.25	31.65±2.69	33.60±0.98	34.69±0.85
**C18:2n6c**	Linoleic acid	21.28±0.41	21.90±0.50	15.74±0.60	23.50±0.95	22.22±1.14	21.34±0.59
**C20:0**	Arachidic acid	0.27±0.20	0.30±0.14	0.37±0.09	0.32±0.08	0.45±0.10	0.31±0.04
**C18:3n6**	-Linolenic acid	0.25±0.07	0.24±0.06	0.19±0.05	0.24±0.10	0.33±0.06	0.22±0.02
**C18:3n3**	Linolenic acid	3.08±0.09	3.14±0.19	2.75±0.34	3.04±0.86	3.85±0.50	4.11±0.80
**C20:1n9**	Eicosenoic acid	1.66±0.50	1.79±0.43	1.84±0.30	2.08±0.39	1.70±0.18	1.94±0.27
**C21:0**	Henicosanoic acid	0.06±0.01	0.06±0.03	0.08±0.04	0.06±0.03	0.13±0.07	0.07±0.02
**C20:2n6**	Eicosadienoic acid	0.91±0.09	0.94±0.09	0.55±0.05	1.01±0.18	0.84±0.08	0.84±0.07
**C22:0**	Docosanoic acid	0.14±0.02	0.17±0.03	0.22±0.03	0.17±0.07	0.30±0.09	0.18±0.03
**C20:3n6**	Dihomo-linolenic acid (DGLA)	0.16±0.05	0.17±0.03	0.22±0.06	0.17±0.07	0.32±0.08	0.26±0.05
**C20:3n3**	Eicosatrienoic acid	0.23±0.03	0.25±0.04	0.32±0.06	0.25±0.08	0.47±0.12	0.47±0.10
**C22:1n9**	Erucic acid	0.20±0.02	0.22±0.03	0.43±0.05	0.25±0.09	0.43±0.09	0.43±0.07
**C20:4n6**	Arachidonic acid	0.61±0.12	0.56±0.10	0.55±0.08	0.47±0.16	0.42±0.06	0.38±0.09
**C23:0**	Tricosanoic acid	0.04±0.01	0.05±0.03	0.06±0.02	0.05±0.03	0.09±0.02	0.05±0.03

**C21:4n3**	Heneicosatetraenoic acid	0.61±0.18	0.68±0.20	0.76±0.12	0.62±0.26	1.23±0.17	0.85±0.19
**C22:2n6**	Docosadienoic acid	0.09±0.03	0.10±0.02	0.13±0.04	0.09±0.02	0.23±0.06	0.16±0.02
**C20:5n3**	Eicosapentaenoic acid (EPA)	4.80±0.63	4.51±0.51	2.46±0.24	3.24±0.71	2.76±0.19	3.06±0.49
**C24:0**	Lignoceric acid	0.09±0.01	0.11±0.03	0.13±0.04	0.11±0.02	0.19±0.03	0.11±0.03
**C24:1n9**	Nervonic acid	0.24±0.20	0.26±0.19	0.50±0.34	0.28±0.19	0.41±0.22	0.37±0.17
**C22:6n3**	Docosahexaenoic acid (DHA)	10.72±0.56	9.50±0.38	6.88±0.50	5.78±0.63	4.82±0.14	5.26±0.29
	**DHA+EPA (%)**	15.51±0.69	14.00±0.53	9.34±0.72	9.02±1.08	7.57±0.30	8.31±0.82
	**r: ω-3 / ω-6**	0.83±0.08	0.76±0.09	0.76±0.07	0.51±0.09	0.54±0.05	0.59±0.09

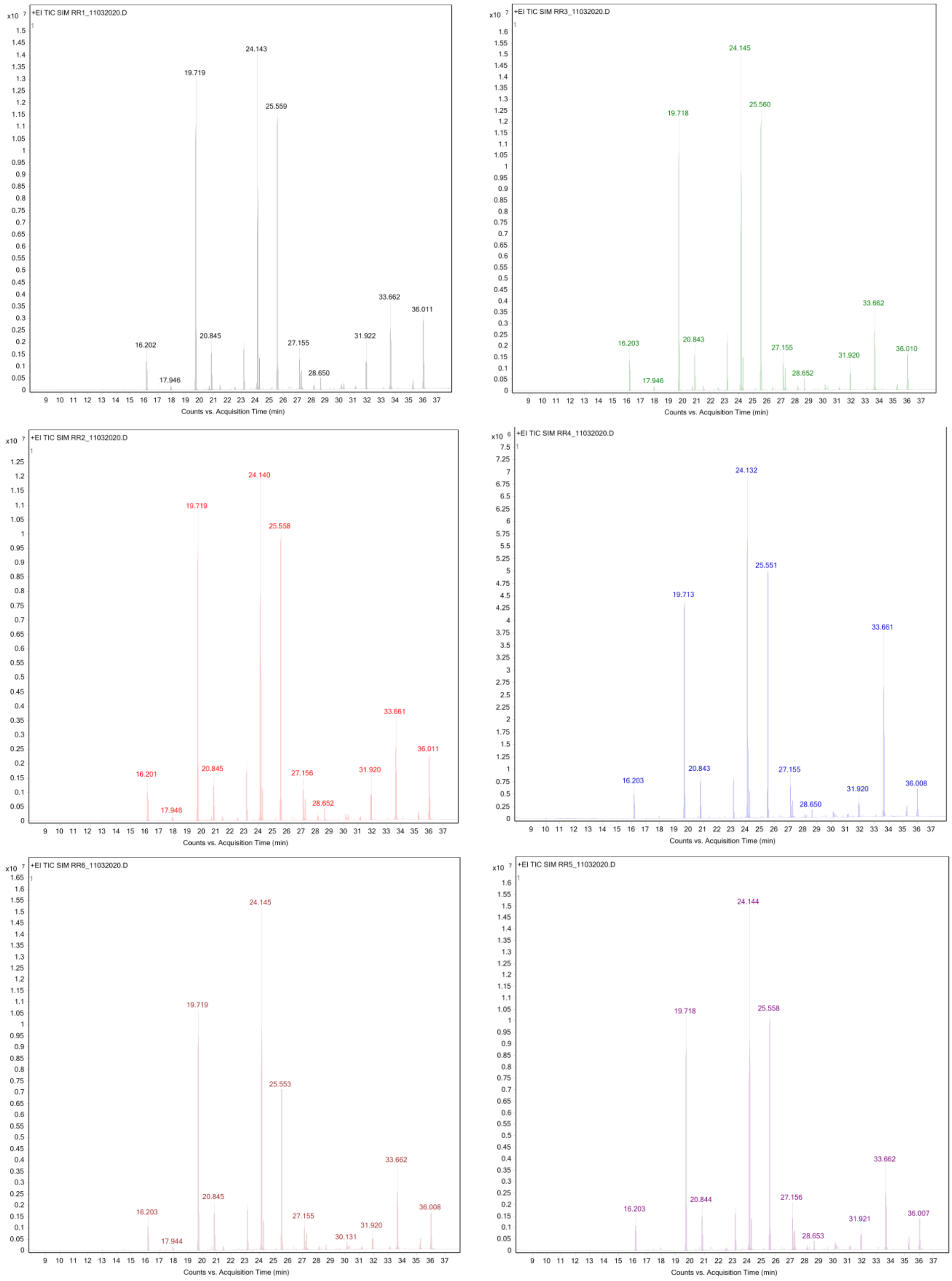

Figure S1. GC-MS chromatograms of oil extracted from Sb_He (top left), Sb_FT (middle left), Sb_Vis (bottom left), Sbass_He (top right), Sbass_FT (middle right) and Sbass_Vis (bottom right).
